# Recent advances in the use of plant growth promoting microorganisms for enhancing micropropagation efficiency

**DOI:** 10.3389/fpls.2025.1699873

**Published:** 2026-01-27

**Authors:** Gurudayal Ram Guru, Pramod W. Ramteke, Csilla Veres, Csaba Vágvölgyi

**Affiliations:** 1 Centre for Tissue Culture Technology, Jacob Institute of Biotechnology and Bioengineering, Sam Higginbottom University of Agriculture, Technology and Sciences, Prayagraj, Uttar Pradesh, India; 2 Department of Molecular Biology and Genetic Engineering, Rashtrasant Tukadoji Maharaj Nagpur University, Nagpur, Maharashtra, India; 3 Department of Biotechnology and Microbiology, Faculty of Science and Informatics, University of Szeged, Szeged, Hungary

**Keywords:** acclimatization, micropropagation, plant growth-promoting microorganisms, rhizobacteria, tissue culture

## Abstract

Micropropagation is an important method within plant biotechnology, allowing the bulk multiplication of high-quality, disease-free plants to occur; however, micropropagation faces several challenges, such as microbial contamination, the expensive chemical products used, and losses occurring during the key acclimatization phase of the micropropagation process. Plant growth-promoting microorganisms (PGPMs) have been shown to ameliorate many of these challenges. These microorganisms support growth and development throughout micropropagation via mechanisms such as nutrient solubilization, phytohormone production and inhibition, and inactivation of pathogens. This review focuses on the potential of the use of PGPMs in the explant initiation, shoot multiplication, rooting, and acclimatization stages and is supported by recent research and the mechanisms of action, challenges, and future perspectives of PGPMs.

## 1 Introduction

Plant biotechnology heavily relies on micropropagation methods to achieve large quantities of valuable crops through precise application of technology. These methods include both *in vitro* and *ex vitro* techniques. *In vitro* micropropagation results in the production of clean, disease free, and genetically identical plants in sterile environments (Debnath et al., 2006). This method is particularly important for producing elite varieties of plants, preserving endangered plant species, or providing plants with phytosanitary certification for global distribution (Micheli et al., 2020).

Pathogen-free planting material may be produced by using *in vitro* techniques, as well as making a clone of an individual plant that will have the same characteristics and multiplying the genetic variability of a particular cultivar in a controlled manner. In contrast, *ex vitro* micropropagation combines both rooting and acclimatization phases under non-sterile but controlled environmental conditions, therefore, *ex vitro* means less use of resources (money) and fewer stresses placed on the plant while still providing a relatively low-cost method to increase root system development, producing a more vigorous plant during acclimatization. Despite these advantages, there are still challenges associated with both methods, *in vitro* systems tend to be more expensive, there is always a risk of somaclonal variation, and plants grown in *in vitro* conditions must be acclimatized to their new environment because they are very sensitive to environmental conditions (Abdalla et al., 2022; Sota et al., 2025), whereas *ex vitro* systems have a greater risk of contamination by microorganisms, and likely do not work well for recalcitrant species.

Micropropagation has many agronomical and industrial uses that go further than solely increasing the amount of certain plants. Examples include: producing virus-free planting materials for crops like bananas and potatos, rapidly multiplying large numbers of their identical clones with superior characteristics (disease-resistance; abiotic stress-resistance), and inducing the production of secondary metabolites (i.e. medically-active chemicals) in plant species with economic importance.

In addition to these uses, micropropagation also increases the rate of return to a plant-breeding programme by providing an easy means of maintaining an elite line and/or allowing rapid testing and development of transgenic cultivars via R&D experiments using plant-tissue culture techniques such as cloning, gene editing, genetic transformation, and regeneration of transferred genes in a new species.

In micropropagation, there are several stages of plant development, including initiation, multiplication, rooting, and acclimation, that are equally important to many different types of plants. The multiplication and rooting phases of development can be particularly challenging for trees and some shrubs due to their recalcitrance and slow growth rates. Acclimatizing and establishing roots is a frequent bottleneck for many herbaceous plants. Medicinal plants often have unique issues related to the production of secondary metabolites and/or metabolite stability during growth. Regardless of plant type, acclimatization is an important factor for all species because it is during this stage that the latest physiological and anatomical adaptations will take place in order to survive when grown in the open.

There are many challenges currently facing the widespread use of modern micropropagation techniques, including phenotypic abnormalities, contamination, and the length of time it takes to establish a micropropagated plant from the laboratory to the field, particularly when the roots of these plants have failed to develop adequately and/or are physiologically stressed.

A possible solution to overcome these limitations may be the use of plant growth-promoting microorganisms (PGPMs) in tissue culture systems. Each stage of micropropagation offers several prospects for these microorganisms to increase plantlet efficacy and mitigate the challenges associated with traditional methods (Cantabella et al., 2022a). PGPMs, which represent a heterogeneous group of advantageous bacteria and fungi, are recognized for their ability to bolster plant growth and vitality through an array of mechanisms, including the synthesis of phytohormones, nutrient solubilization, suppression of pathogens, and alleviation of environmental stresses (Gangwar et al., 2017). Genera that are known to have received considerable research attention, such as *Pseudomonas, Bacillus, Azospirillum, Rhizobium*, and *Trichoderma*, have already demonstrated their ability to support plant growth in both natural and cultivated ecosystems (Rai et al., 2020). Over the past few years, significant attention from academic circles has been devoted to the practice of integrating PGPMs into micropropagation systems (Yablonskaya et al., 2016; Castro et al., 2019; Modi and Kumar, 2021). These PGPMs provide an economical option for increasing plantlet growth and survival, lowering microbial contamination, and reducing the use of synthetic phytohormones. These traits make them a potent means for improving the efficiency and sustainability of micropropagation technologies (Chouhan et al., 2021).

**Figure 1:**
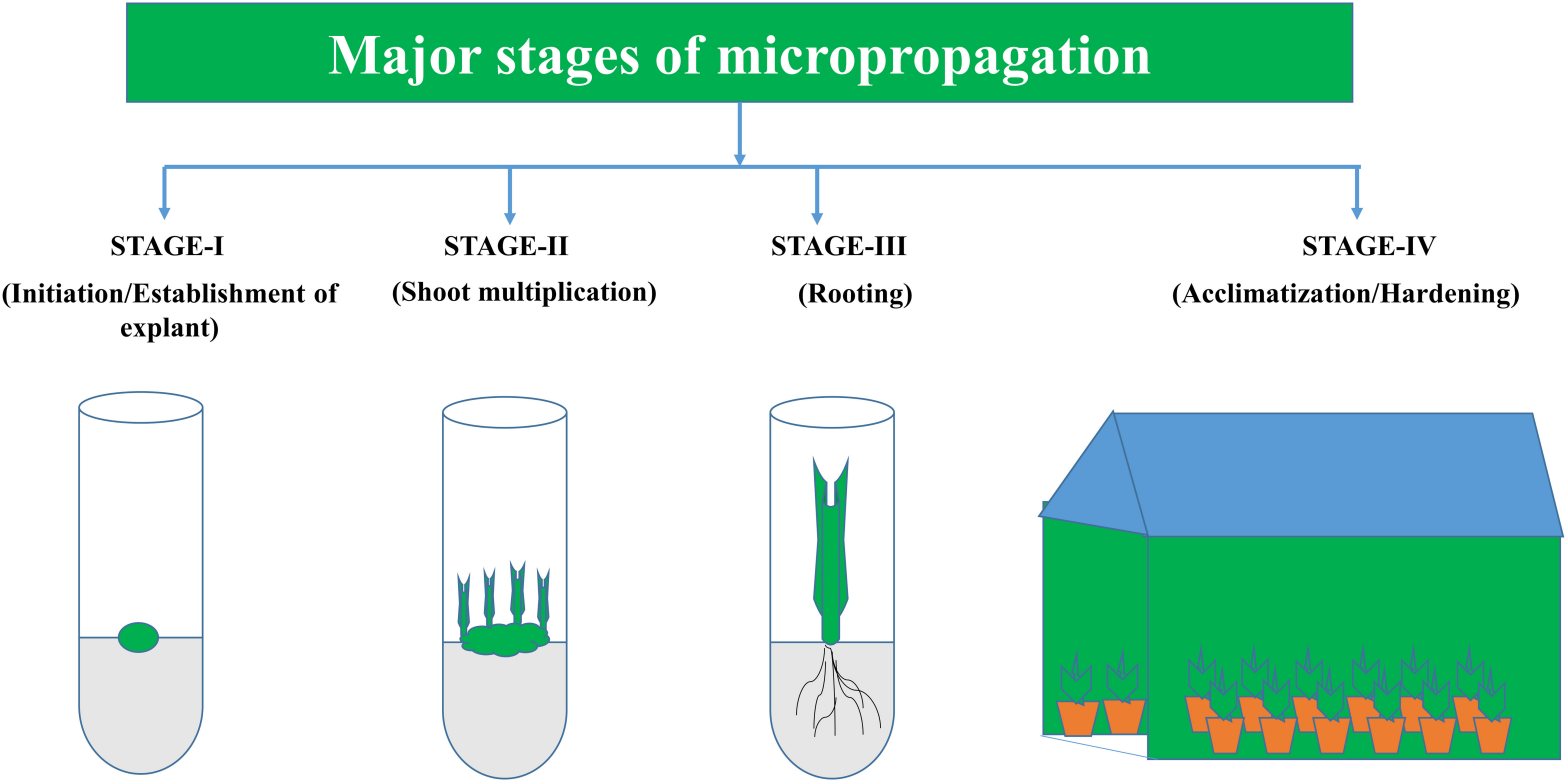
Major stages of micropropagation.

Within the context of micropropagation, plant growth-promoting microorganisms can be realistically studied in four major stages (**Figure 1**): (1) explant initiation, (2) shoot proliferation, (3) root formation, and (4) hardening and acclimatization. Each phase has specific needs and challenges that need to be addressed to obtain viable plantlets (Torres, 1989).

The beneficial impacts of PGPMs are exercised through numerous mechanisms, which can be broadly classified into direct and indirect pathways (Gamalero and Glick, 2011; Glick, 2012). The direct pathways involve the synthesis of plant growth regulators that influence many physical activities in a plant. In addition, PGPMs improve the supply of nutrients through phosphate solubilization, nitrogen fixation, and the production of siderophores (Mohanty et al., 2021). Indirect mechanisms include overgrowing pathogens by producing suppressive agents, such as antibiotics, hydrogen cyanide (HCN), and lytic enzymes. PGPMs also compete with pathogens, demonstrating the protection of plantlets against them (Chouhan et al., 2021) and assisting plants in coping with drought, salinity, and heavy metal poisoning by increasing plant tolerance to abiotic stress and oxidative injury (Kumar et al., 2019).

PGPMs are promising candidates for improving micropropagation systems because of their advantages. Their efficacy is often strain-specific, depending on the plant species, type of tissues on which the bacterial strain is applied, and environmental conditions. Consequently, plant growth-promoting microbial strains can be more or less effective. Moreover, some of these high-density PGPMs compete with plant tissues for nutrients in culture media or undergo secondary contamination, necessitating careful optimization of their concentration and application methods. A second caveat is the absence of standardized protocols for integrating PGPMs into tissue culture systems, which limits their commercial adoption. This is a challenge, and more research and development are needed to optimize the use of PGPMs in micropropagation and for maximum benefit.

Microbial inoculation during acclimatization is beneficial for improving the survival and stress tolerance of plants in micropropagation. However, limitations to this approach include variations in how consistently microorganisms colonize plant roots, potential contamination during the inoculation process and the regulation of microbial inoculants. A different approach is to use the metabolites of microorganisms as biostimulants and bioprotectors without introducing the microorganisms themselves (Parnell et al., 2016; Emmanuel and Babalola, 2020; Morcillo et al., 2022; Ansari et al., 2023; Kumar et al., 2024; Li et al., 2025).

Furthermore, metabolites produced by microbes, such as volatile organic compounds (VOCs) and phytohormones, have been demonstrated to enhance root development, confer stress tolerance to micropropagated plants, and influence the regulation of secondary metabolites within the tissues of these plants. Consequently, leveraging metabolites as a substitute for microbial inoculation presents a more favorable option for the advancement of standardized and safer biostimulation technologies in future micropropagation.

## 2 Action mechanisms of PGPMs in micropropagation

**Figure 2:**
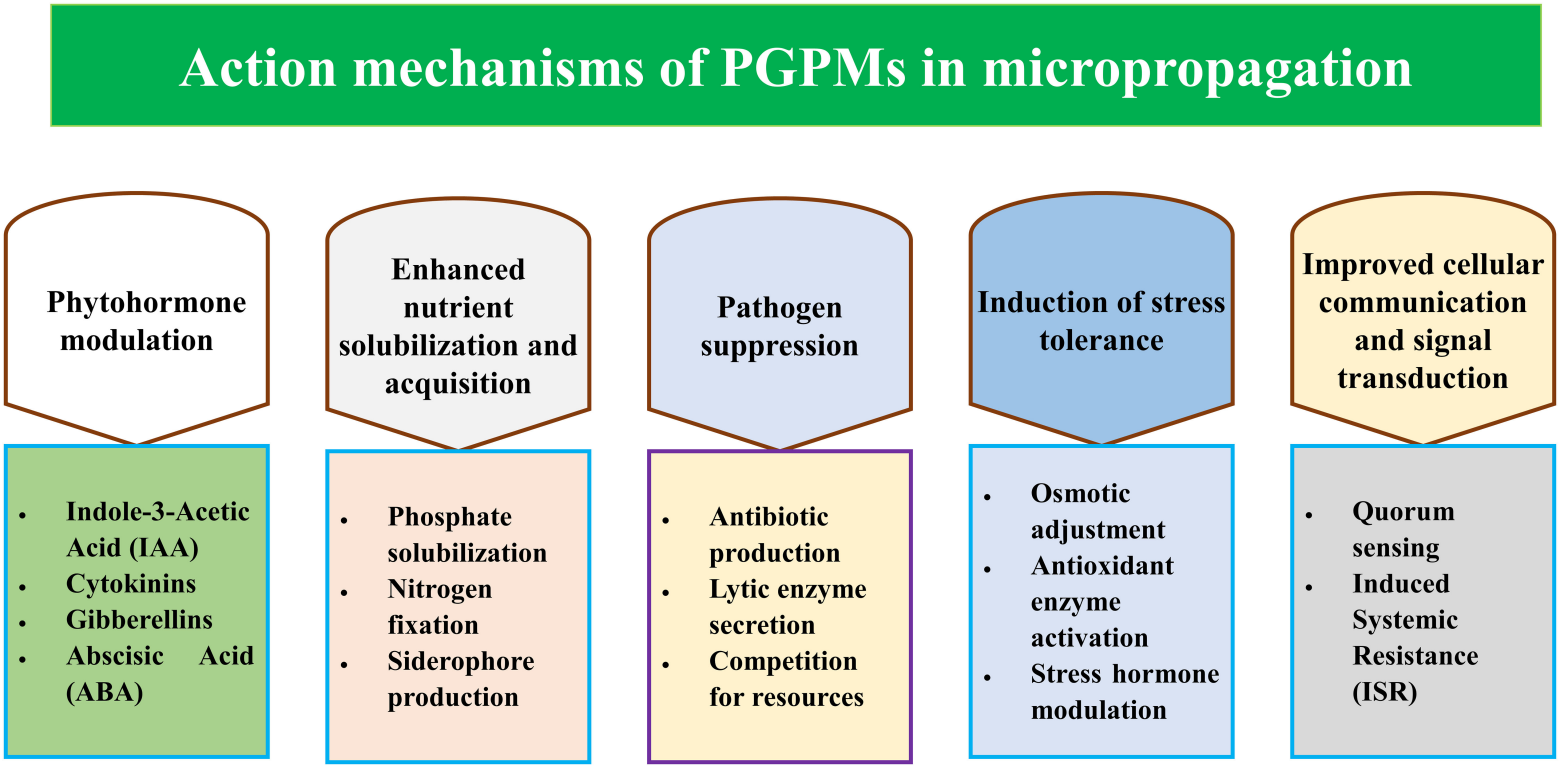
Action mechanisms of PGPMs in micropropagation.

PGPMs are known for their ability to stimulate plant growth and health throughout various phases of development. Their use in micropropagation presents new methods for increasing the efficacy, sustainability, and profitability of tissue culture of plants. PGPMs can benefit the performance of plantlets through increased nutrient uptake, increased phytohormone activity, increased disease resistance, improved tolerance to stress and improved cell signaling (**Figure 2**). These benefits make PGPMs crucial in addressing challenges faced in micropropagation, such as their dependence on synthetic growth regulators, microbial contamination, and relatively high mortality rates during acclimatization.

A major mechanism by which PGPMs can stimulate the growth of plantlets during micropropagation is their ability to produce phytohormones. Plant regulators are paramount in the regulation of critical physiological processes, such as cell division, elongation, differentiation, and stress response. The phytohormones most often studied related to these microorganisms are indole-3-acetic acid (IAA), cytokinins, gibberellins, and abscisic acid (ABA). IAA is an auxin and is critical for the elongation of cells, root initiation, and the formation of lateral roots (Ghosh et al., 2013). PGPMs can produce IAA directly or convert tryptophan, which is a precursor to IAA, which is commonly provided in plant tissue culture media (Ahmad et al., 2005). It is important for root development and for enhancing the uptake of other nutrients, which are important for the increased survival of plantlets during acclimatization (Sobczyk and Szzwejkowska, 2015). Cytokinins are important for cellular division and for shoot initiation (Kiba and Sakakibara, 2010). PGPMs that produce cytokinins support shoot multiplication by imitating the effects of synthetic cytokinins used in plant tissue culture. These microorganisms help to extend the cell cycle, allowing a relatively large number of shoots to emerge and elongate in various species (de Garcia Salamone et al., 2005). Gibberellins are important in the regulation of stem elongation (Jones and Kaufman, 1983) and seed germination (Ritchie and Gilroy, 1998). PGPMs can produce gibberellins that promote internode elongation, making these microorganisms useful in growing shoots during the shoot multiplication stage of micropropagation (Gutierrez and Probanza, 2000). While gibberellins are often associated with the regulation of stress responses, PGPMs that produce abscisic acid (ABA) assist in stomatal regulation, which is especially beneficial in the hardening of plantlets during the *ex vitro* transition (Hronková et al., 2003). They provide an organic source of phytohormones, thus minimizing the use of synthetic plant growth regulators and increasing the sustainability and economic feasibility of micropropagation systems.

Nutrient availability in the culture medium is another significant factor in plantlet growth and development during micropropagation. PGPMs promote nutrient absorption by fixing atmospheric nitrogen, mobilizing micronutrients, and solubilizing macronutrients. Phosphorus is one of the most important macronutrients and is often in an insoluble form that is almost impossible for a plant to utilize. The microorganisms that solubilize phosphate produce organic acids, including citric acid and gluconic acid, which can convert insoluble forms into soluble forms. Increasing the availability of phosphate promotes root and shoot development (Vyas and Gulati, 2009; Amalraj et al., 2012). Even though nitrogen is another necessary nutrient for plant growth, it is often scarce in tissue culture media. Nitrogen-fixing PGPMs, such as *Azospirillum* and *Rhizobium*, convert dinitrogen gas into ammonia, which is easily used by plants (Bergersen and Gibson, 1978; Elmerich et al., 1987). This biochemical transformation is particularly advantageous during the shoot multiplication and rooting phases. Iron, a micronutrient, is critical for plant metabolic processes but tends to be found in forms inaccessible to plants. PGPMs produce siderophores, which are high-affinity iron-chelating molecules that bind to and make iron available to plantlets. Siderophore-producing PGPMs are important for chlorophyll synthesis and general plantlet health (Pahari et al., 2017). Intensive research is underway to examine the effects of these microorganisms on providing nutrients and optimizing growth with minimal reliance on expensive nutrient-rich culture media.

Micropropagation can be inefficient and poses a risk to precious plant tissue because of damage caused by microbial pathogens. PGPMs have been proven to cure organisms associated with pathogenic issues by combating pathogenic organisms. Other studies have shown that certain growth promoting microorganisms, such as *Bacillus subtilis* and *Pseudomonas fluorescens*, produce antifungal and antibacterial metabolites that serve as antibiotics (Ganeshan and Kumar, 2005; Quan et al., 2010; Majumder et al., 2014). *Bacillus subtilis* has also been shown to produce membranolytic compounds, such as surfactin and iturin, indicating that bacterial and fungal pests are attacked and their cell membranes are disrupted (Yang et al., 2022). These microorganisms also produce lytic enzymes, such as chitinases, glucanases, and proteases, which work on the cell membranes of fungal and bacterial pathogens (Bateman and Basham, 1976; Anitha and Rabeeth, 2010).

*Trichoderma* spp. are among the most prominent biocontrol agents for complex fungi through tissue culture (Kaur and Kaur, 2022). PGPMs are known for improving pathogen efficiency, which increases the likelihood that the area will be contaminated while competing with plant pathogens for nutrients and space. This phenomenon is most commonly observed during the explant initiation and acclimatization stages (Chouhan et al., 2021).

## 3 Effect of PGPMs on major stages of micropropagation

**Figure 3:**
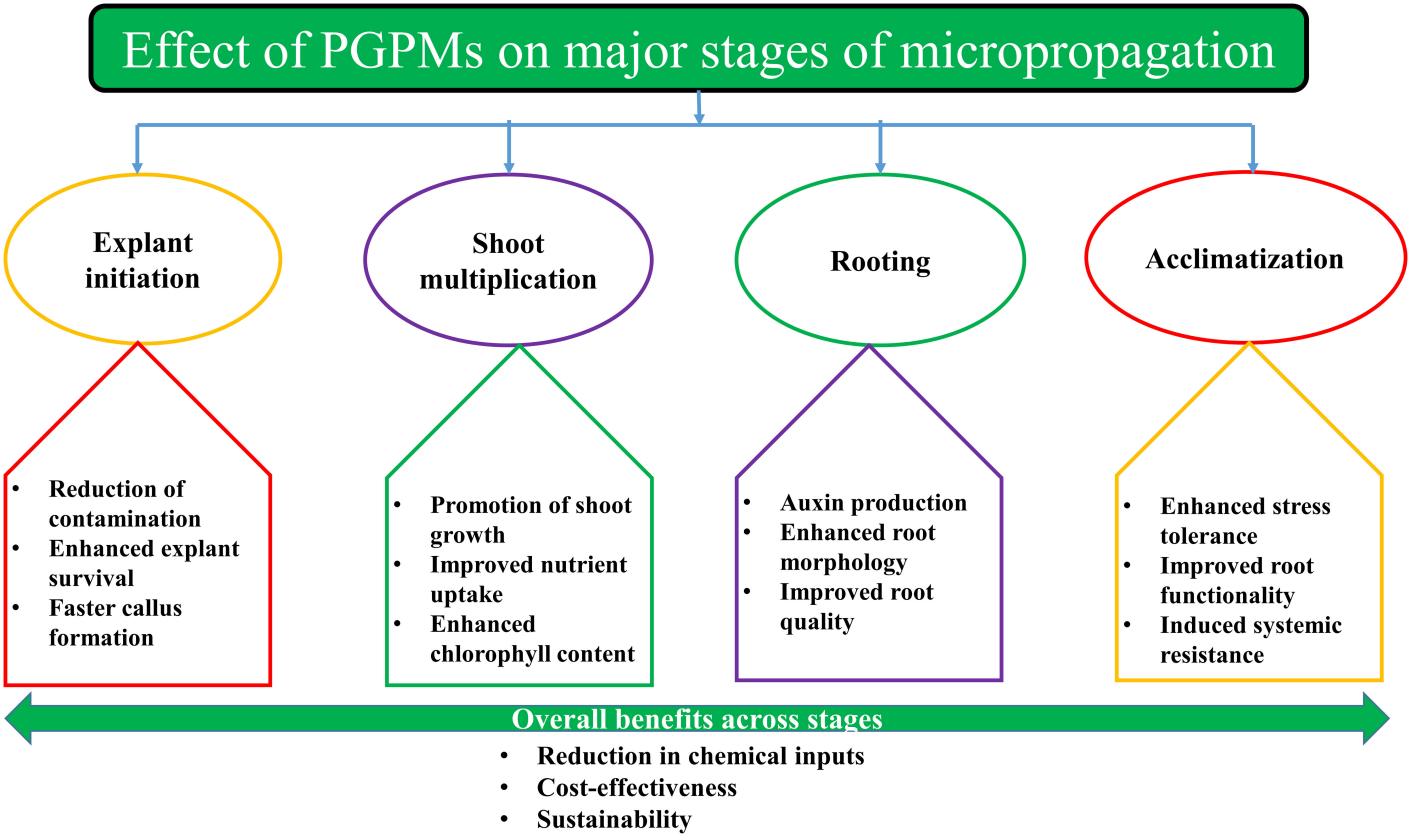
Effect of PGPMs on major stages of micropropagation.

The addition of PGPMs has enabled significant advancements in plant tissue culture, addressing the issues inherent in traditional micropropagation techniques. Micropropagation is a complex process and requires precision and ideal environmental conditions in each phase. They play an important role in improving the efficiency and success at each stage of micropropagation, from the explant initiation stage to the final acclimatization stage (**Table 1**). PGPMs influence various stages by promoting the production of phytohormones, increasing the availability of nutrients, inhibiting pathogens, and increasing plantlet resistance to biotic and abiotic stresses (**Figure 3**).

**Table 1:** Effect of plant growth promoting microorganisms on major stages of micropropagation.

S. no.	Plant species	PGPM strain(s)	Type of PGPMs	Micropropagation stage(s)	Effect on micropropagation	Reference
1.	*Allium sativum* cv. “Gigante Roxo”	*Enterobacter cloacae* M19B *Burkhloderia cepacia* CCMA0056	Endophytic bacteria	Initiation stage	Enhanced seedlings growth, chlorophyll content and carotenoid content	Costa Júnior et al. (2020).
2.	Jaboticaba (*Plinia peruviana*)	*Stenotrophomonas* sp.	Endophytic bacteria	Initiation stage	Enhanced rate of seed germination.	Queiroz et al. (2020).
3.	*Bromus auleticus* (Trin.)	*Epichloë* spp.	Endophytic Fungi	Initiation/Multiplication/Rooting/Acclimatization	Enhanced *in vitro* germination, callus induction, and plant regeneration percentages, as well as promoted greater growth of the regenerated plantlets	Regalado et al. (2018)
4.	Oil palm (*Elaeis guineensis* Jacq.).	*Herbaspirillum seropedicae* strain Z78 (ATCC 35893)	Rhizobacteria	Multiplication	Promoted growth of *in vitro* calli and embryogenic calli of oil palm under symbiosis conditions.	Lim et al. (2016)
5.	*Chrysanthemum x grandiflorum* (Ramat.) Kitam ‘Ludo’ *Gerbera jamesonii* ‘Kormoran’;	*Paenibacillus* *glucanolyticus*	Bacteria	Multiplication and Rooting	Enhanced the number and length of shoots.	Zawadzka et al. (2014)
6.	*Chrysanthemum* x *grandiflorum* (Ramat.) Kitam ‘Ludo’; *Gerbera jamesonii* ‘Kormoran’; *Hosta* ‘Paradigm’; *Rosa* L. ‘White Gem’	*Curtobacterium pusillum*	Bacteria	Multiplication and Rooting	Stimulated axillary shoot formation.	Zawadzka et al. (2014)
7.	*Gerbera jamesonii* ‘Kormoran’ *Hosta* ‘Paradigm’ *Chrysanthemum* x *grandiflorum* ‘Ludo’;	*Methylobacterium* *extorquens*	Bacteria	Multiplication and Rooting	Showed significant benefits for rooting and shoot multiplication in gerbera and hosta.	Zawadzka et al. (2014)
8.	*Fouquieria* sp*lendens*	*Staphylococcus pasteuri strain* Fs3,	Endophytic bacteria	Multiplication	Protected callus against cadmium under *in vitro* conditions.	Espinoza-Mellado et al. (2020)
9.	UCB-1 pistachio	*Pseudomonas* *fluorescens* VUPF5 and *Bacillus subtilis* VRU1	Rhizobacteria	Multiplication and Rooting	Increased the growth and proliferation and improved elongation and rooting in tissue culture.	Pour et al. (2019)
10.	Potato (*Solanum tuberosum* L. cv. Nevsky) microplants	*Azospirillum brasilense* Sp245 or *Ochrobactrum cytisi* IPA7.2.	Rhizobacteria	Rooting	Showed growth-stimulating effect.	Arkhipova et al. (2020)
11.	Potato (*Solanum tuberosum* L.)	*Ochrobactrum cytisi* IPA7.2	Bacteria	Rooting	Promoted the growth of potato microplants.	Burygin et al. (2019)
12.	Pyrus (Py170 and Py12) rootstocks and Prunus RP-20 rootstock	*Cladosporium* *Ramotenellum* PGP02, *Phoma* spp. PGP03, *P. oryzihabitans* PGP01	Rhizosphere	Rooting	Enhanced rooting percentage and plant growth.	Cantabella et al. (2021)
13.	*Arabidopsis* *thaliana*	*Aeromonas punctata* PNS-1	Rhizobacteria	Rooting	Increased primary root length and lateral root density.	Iqbal and Hasnain (2013)
14.	*Pyrus communis* L. (cv Dar Gazi microcuttings)	*Pantoea agglomerans*	Bacteria	Rooting	enhanced adventitious root formation, promoting earlier root emergence and improved root architecture.	Luziatelli et al. (2020)
15.	*Olea europea* L. (cv. *Rowghani*)	*Pseudomonas* *fluorescent* P19 or P21	Rhizobacteria	Rooting	Induced almost two times increase in number and length of roots per explant.	Peyvandi et al. (2010)
16.	*Prunus avium* ‘Achilleus’ *P. avium* ‘Fama’	*Microbacterium testaceum* *Rhodopseudomonas* spp.	Endophytic bacteria	Rooting	Increased rooting percentage and number of roots per shoot	Quambusch et al. (2014)
17.	*Prunus avium*	*Rhodopseudomonas palustris* N-I-2 and *Microbacterium* *testaceum* D-I-1	Endophytic bacteria	Rooting	Promoted rooting of two difficult-to-propagate *P. avium* genotypes	Quambusch et al. (2016)
18.	*Arabidopsis thaliana*	*Pseudomonas* spp	Rhizobacteria	Rooting	Stimulated host’s endogenous programs related to primary root, LR, and RH development.	Zamioudis et al. (2013)
19.	*Handroanthus impetiginosus* ‘pink lapacho’	*Stenotrophomonas* L20, *Advenella* sp L21, *Sphingobacterium* sp. L22, *Brevundimonas* L23, and *Bacillus* L24 and L25	Rhizobacteria	Rooting.	Prevented plant loss and reduced production costs by minimizing synthetic auxins requirements.	Yarte et al. (2022)
20.	Photinia (*Photinia* × *fraseri* Dress),	*Azospirillum brasilense* and *Azotobacter* *chroococcum*	Rhizobacteria	Rooting	Induced earlier rooting of shoots.	Larraburu et al. (2007)
21.	Banana (*Musa* cv. Red Banana)	*Pseudomonas fluorescens* Pf1 and *Bacillus subtilis* strains 10 and 56	Endophytic and Rhizospheric bacteria	Rooting	Enhanced plant growth and disease resistance.	Kavino and Manoranjitham (2018)
22.	H*plleborus	*Burkholderia* *phytofirmans* PsJN	Bacteria	Rooting and Acclimatization	Increased the *in vitro* rooting percentage and number of roots per shoot. Enhanced acclimatization in greenhouse condition.	Orlikowska et al. (2017)
23.	Marubakaido apple rootstock	Rhizobia	Rhizobacteria	Rooting and acclimatization	Enhanced the rooting and acclimatization of micropropagated Marubakaido apple rootstock.	Muniz et al. (2013)
24.	Tea (*Canellia sinensis*)	*Trichoderma harzianum*, *Azospirillum brasilense*, *P. fluorescens*	Fungi and Bacteria	Rooting and Acclimatization	Enhanced acclimatization success, promoting growth and systemic resistance.	Thomas et al. (2010)
25.	Cavendish banana	*Pseudomonas fluorescens, Bacillus pudita, Bacillus amyloliquefaciens* and *Bacillus pumilus*	Rhizospheric and Endophytic bacteria	Rooting and Hardening	Enhanced plant growth parameters of tissue culture banana plantlets under greenhouse condition.	Mon et al. (2021)
26.	Banana cv. Prata Anã and BRS Princesa	*Bacillus cereus* strains	Endophytic bacteria	Acclimatization	Reduced acclimatization period and improved root growth	Araújo et al. (2023)
27.	Sugarcane variety TUC 03-12	182-*Bacillus* and 336-*Pseudomonas*	PGPB	Acclimatization	Increased survival rates during acclimatization	Michavila et al. (2022)
28.	Banana variety "Grand Naine”	*Azotobacter, Azospirillum, Rhizobium, Pseudomonas*, PSB, and VAM	Bacteria and VAM	Acclimatization	Enhanced survival and growth of plants. Consortium treatment improved root, shoot, and nutrient uptake.	Panigrahi et al. (2016)
29.	Banana cv. ‘Prata Catarina’	*Bacillus* sp. strains	Bacteria	Acclimatization	Increased nitrogen, potassium, and magnesium content in leaves and roots	Rodrigues et al. (2022)
30.	Potato (*Solanum tuberosum* L. cv. Nevsky)	*Azospirillum baldaniorum* Sp245	Bacteria	Acclimatization	Enhanced antioxidant protection and survival under aeroponic conditions	Tkachenko et al. (2023)
31.	Blackberry microclones (*Rubus fruticosus* L.)	Actinobacteria isolates	Bacteria	Acclimatization	Increased survival rate, shoot length, node numbers, and leaf area	Tytarenko et al. (2023a)
32.	*Rubus fruticosus* L. and *Paulownia tomentosa* Steud.	*Enterococcus italicus* ONU547	Bacteria	Acclimatization	Improved survival rates, shoot length, node numbers, and leaf area	Tytarenko et al. (2023b)
33.	Pear (*Pyrus communis* L.) and Nectarine (*Prunus persica* L. cv. Nectarine)	*Cladosporium ramotenellum* strain PGP02 and *Phoma* spp. strain PGP03, and *Pseudomonas oryzihabitans* PGP01	Rhizobacteria and Rhizofungi	Acclimatization	Enhanced the acclimatization efficiency to soil in those crosses with poor acclimation efficiencies.	Cantabella et al. (2020)
34.	*Mentha* sp*icata* L.	*Trichoderma* *asperellum* and *Bacillus subtilis*	Endophytes	Acclimatization	Improved plant survival and growth. Decreased the incidence of *Rhizoctonia* sp., and improved the contents of specialized metabolites.	Castro-Restrepo et al. (2022)
35.	Banana plantlets in secondary hardening stage	*P. fluorescens* Pf1 and CHA0 *Pseudomonas* spp. EPB22 *Bacillus* spp. EPB5)	PGPR and PGPE	Acclimatization	Reduced Banana bunchy top disease by induction of systemic resistance.	Harish et al. (2008)
36.	*Albizia amara*	*Pseudomonas* *fluorescens* and *Trichoderma viride*	Endophytes (both fungi and bacteria)	Acclimatization	Enhanced plant survival.	Indravathi and Suresh Babu (2019)
37.	*Musa acuminata* Colla AAA, cv. ‘Grande Naine’ and TMBx 5295-1	*Bacillus* spp.	PGPR	Acclimatization	Enhanced plant development and health.	Jaizme-Vega et al. (2004)
38.	*Vernonia* *divergens*	*Piriformospora indica*	Fungus	Acclimatization	Enhanced the survival rate and growth of root, shoot andnleaf of the regenerated plants.	Kumar et al. (2014)
39.	Tea	*B. subtilis* *Bacillus* spp. *Pseudomonas corrugata* 1 *Pseudomonas corrugata* 2	Rhizobacteria	Acclimatization	Higher survival rate in greenhouse conditions.	Pandey et al. (2000)
40.	Banana plantlets (*Musa* spp. cv Grand Naine) in primary and secondary hardening stages	*B. subtilis* strains PP and CL3	Bacteria	Acclimatization	Induced defence enzymes and pathogenesis-related proteins.	Rajamanickam et al. (2018)
41.	Banana (*Musa acuminata* Colla AAA, cv. ‘Grande Naine’)	Rhizobacteria consortium of *Bacillus* spp. (with AMF *Glomus manihotis*)	Rhizobacteria and arbuscular mycorrhizal fungi (AMF)	Acclimatization	Combined inoculation enhanced banana growth and nutrition. Increased leaf mineral content without adverse effects.	Rodríguez-Romero et al. (2005)
42.	*Musa acuminata* cv. Grand Naine	*Pseudomonas putida*, *Pseudomonas flourescens*, and *Bacillus* sp.	Plant associated bacteria	Acclimatization	Improved growth parameters with a reduced hardening period.	Suada et al. (2015)
43.	*Picrorhiza kurrooa*	*Bacillus megaterium*, *B. subtilis* and *Pseudomonas corrugata*	PGPR	Acclimatization	Improved survival and growth parameters.	Trivedi and Pandey (2007)
44.	Fruit rootstocks: Mr. S 2/5plum (*Prunus cerasifera* x *P.* sp*inosa)*, GF 677 hybrid (*Prunus persica* x *P. amigdalus*), and MM 106 apple (Northen Spy x M1)	*Azospirillum brasilense* Sp245	Rhizobacteria	Acclimatization	Showed higher vigor, consistent with a wider leaf area, in the inoculated plantlets	Vettori et al. (2010)
45.	*Gloriosa superba* L.	*Glomus mosseae* , *Acaulospora laevis*	AMF	Acclimatization	Enhanced plant growth and higher colchicine content.	Yadav et al., 2013).
46.	Banana cv. Robusta (AAA)	*Pseudomonas fluorescens* Pf1, CHA0 and *Bacillus subtilis* EPB22	*Endophytic bacteria*	Primary and Secondary hardening	Protected young plantlets from transplanting stresses in field.	Kavino et al. (2007)
47.	Blackberry plants (*Rubus glaucus* L.)	AMF: Glomus sp. (GEV02) PGPR: Pseudomonas migulae (Pf014) *Bacillus amyloliquefaciens* (Bs006)	AMF and PGPR	Hardening and acclimatization	Improved survival (≥80 %) in the stages of hardening and acclimatization.	Moncada et al. (2015)

### 3.1 Explant initiation stage

The initiation stage of micropropagation involves sterilizing and placing explants in nutrient media under aseptic conditions. The initiation stage is one of the more critical stages of the micropropagation culture process. The main difficulties are contamination and stresses that lead to plant death, thus affecting the total number of explants. PGPMs are entirely natural and may act as environmentally friendly means of addressing these complications. One of the main benefits of these microorganisms at the initiation stage is a reduction in microbial contamination (Cantabella et al., 2022a). Microbial contaminants, such as bacteria and fungi, often limit explant survival, leading to significant losses (Leifert and Waites, 1994; Benjama and Charkaoui, 1997). Certain PGPMs have antifungal and antibacterial properties, which limit microbial contamination and support better growth and survival of explants during the start of micropropagation (Nowak, 1998). In addition, they also suppress pathogen growth through the production of antibiotics, hydrogen cyanide (HCN) and lytic enzymes with antimicrobial capabilities (Navarro et al., 2019; Ali et al., 2020). They prevent microbial contamination from pathogens by competing for nutrients and space.

Queiroz et al. (2020) reported that *Bacillus* sp. and *Stenotrophomonas* sp. produced highest levels of indolic compounds (27.41 μg mL^−1^) and significantly increased germination of jaboticaba seed (97.34%). Regalado et al. (2018) showed that the presence of *Epichloë endophytes* in *Bromus auleticus* seeds significantly improved *in vitro* culture conditions and enhanced regeneration of plants. Seeds infected with endophytes (E+) had higher germination (82% vs. 57%), callus induction (72% vs. 37%), and plant regeneration (89% vs. 13%) compared to seeds free from endophytes (E−). Salotti et al. (2023) assessed the impact of plant growth-promoting bacteria on the micropropagation of *Jacaranda mimosifolia*. Both *Methylobacterium* sp. L10 and *Stenotrophomonas* sp. L20 improved plant performance at all stages when biotized and effectively inhibited fungal contamination during establishment and demonstrated strong biocontrol potential. Additionally, PGPMs can manage the population of endophytes during micropropagation by adjusting the pH of the culture medium. Cantabella et al. (2022b) evaluated the GreenTray^®^ temporary immersion system (TIS) bioreactor for Prunus Rootpac 20^®^ micropropagation, as well as its interactions with *Pseudomonas oryzihabitans* PGP01 and *Cladosporium ramotenellum*. Alterations in the pH in the GreenTray^®^ TIS bioreactor were showed to affect microbial growth.

The sterilization and transfer of explants to artificial media may induce oxidative stress and initiate tissue damage (Uhm et al., 2013). PGPMs mitigate stress by producing antioxidants and activating stress-responsive pathways within explants (Enespa et al., 2020). They promote the production of antioxidant systems such as superoxide dismutase (SOD) and catalase (CAT) and alleviate oxidative damage, thus generally increasing explant survival rates (Chouhan et al., 2021).

Overall, PGPMs are beneficial for plant growth and survival through micropropagation by improving nutrient absorption, encouraging the plant to strengthen its defenses and reducing microbial contamination and transplant shock at the early stages of *in vitro* culture (Soumare et al., 2021).

### 3.2 Shoot multiplication stage

The purpose of the shoot multiplication phase is to proliferate multiple shoots from the explant. This phase historically relied on the use of synthetic cytokinins such as benzylaminopurine (BAP) for the initiation and proliferation of shoots. However, PGPMs naturally synthesize cytokinin-like substances that mimic the physiological action of synthetic cytokinin to promote the initiation and proliferation of shoots.

García de Salamone et al. (2001) demonstrated that *Pseudomonas fluorescens* G20–18 produced cytokinins beneficial for plant growth. Mutants with reduced cytokinin production confirmed its functional role. Addition of adenine increased cytokinin synthesis, emphasizing its role in PGPR-mediated growth promotion. Lim et al. (2016) explored an artificial symbiosis between *Herbaspirillum seropedicae* Z78 and *in vitro* oil palm calli. The study revealed that diazotroph mediated biotization could be a promising approach for improvement in micropropagation of oil palm. According to Zawadzka et al. (2014), the incorporation of bacterial strains *Paenibacillus glucanolyticus, Curtobacterium pusillum* and *Methylobacterium extorquens* into tissue culture had a beneficial effect on the number of shoots produced in chrysanthemum, gerbera, hosta and rose. *C. pusillum* had a consistent positive impact on the number of shoots produced from axillary buds. All of these bacteria produced indole-3-acetic acid (IAA) and assimilated nitrogen, indicating that they may have potential use as beneficial inoculants in micropropagation.

Espinoza-Mellado et al. (2020) explored the use of two endophytic bacteria to “biotize” callus from *Fouquieria* sp*lendens*, and evaluated how their effects differed when grown under cadmium stress. *Staphylococcus pasteuri* Fs3 exhibited a greater degree of protection and increased callus growth compared to *Kocuria rhizophila* Fs7. The authors suggested that there was genotypic specificity between plant and bacterial hosts. They also concluded that callus age and quality were significant factors that influenced the degree of biotization success. As such, *S. pasteuri* Fs3 may be used as a biostimulant/protective inoculant in tissue culture for plants stressed by heavy metals.

Pour et al. (2019) studied how to enhance the efficiency of UCB-1 pistachio micropropagation using nanoencapsulated PGPR. The PGPR used were *Pseudomonas fluorescens* and *Bacillus subtilis*. They found that the use of nanoencapsulated metabolites significantly improved shoot proliferation, and that nanoformulations provided the significant impact. The authors also noted that the use of direct inoculation techniques caused explant desiccation, and emphasized the need for controlled delivery systems for future studies.

The PGPMs increase the multiplication rate of plant shoots during micropropagation by acting as biofertilizers, providing nutrients, producing plant growth regulators, and suppressing pathogens, which collectively improve plant growth during *in vitro* culture (Cantabella et al., 2022a).

### 3.3 Rooting stage

Rooting is the most crucial stage in micropropagation, as a functioning root system sets the basis for the successful acclimatization of plantlets in *ex vitro* environments. This stage often relies on synthetic auxins, such as indole-3-butyric acid (IBA), to stimulate rooting. Nevertheless, PGPMs present a natural and effective substitute. In particular, blue–green algae-associated PGPMs, such as *Pseudomonas fluorescens* and *Azospirillum brasilense*, contribute to the formation and growth of roots. These microbes synthesize IAA, stimulating root tissue division and differentiation (Přikryl et al., 1985; Patten and Glick, 2002; Reetha et al., 2014; Park et al., 2021). In addition, the application of PGPRs micropropagation aids in root system development in terms of hormonal concentrations, root system architecture, and nutrient uptake. These bacterial sources of hormones include auxins and cytokinins that promote root hair proliferation and lateral root growth, thereby improving nutrient uptake (Turan et al., 2021).

PGPMs play a critical role in rooting during micropropagation by synthesizing plant growth regulators, such as auxins, which stimulate lateral root development and root plasticity to improve nutrient uptake, which is essential to the successful rooting of tissues or seedlings grown *in vitro* (Cantabella et al., 2022a). These growth-promoting microbes also increase not only the quantity of roots but also their length, diameter, and branching structure. The contributions to root development are critical, especially for the later stages of development, since roots are responsible for the uptake of both nutrients and water (Chouhan et al., 2021; Grover et al., 2021).

Zawadzka et al. (2014) found that bacterial strains isolated from tissue cultures, specifically *Paenibacillus glucanolyticus, Curtobacterium pusillum*, and *Methylobacterium extorquens*, all have positive effects on the rooting of chrysanthemum, gerbera, hosta, and rose. Cantabella et al. (2021) reported that in the *Pyrus* Py12 rootstock, the rooting ability increased from 56.25% with the use of IBA to 100% with the use of fungal inoculation. The production of IAA by all three fungi confirmed their role as plant hormones. *P. oryzihabitans* PGP01 and *C. ramotenellum* PGP02 had a significant improvement in the root growth of both *Pyrus* and *Prunus* rootstocks and indicated their potential as natural biostimulants to replace chemical auxins in tissue culture systems. Iqbal and Hasnain (2013) experimented on *Arabidopsis* seedlings and reported that *Aeromonas punctata* strain PNS-1 had the potential to be used as a plant growth-promoting rhizobacteria (PGPR) and might affect the way roots form by way of hormones and enzyme activity. *Pantoea agglomerans* C1, was shown to induce adventitious roots in pear (*Pyrus calleryana*) microcuttings through modulation of auxin-related gene expression. Exometabolites produced by *Pantoea agglomerans* C1 were more effective in promoting root emergence from pear micro-cuttings than synthetic auxins (IBA) and have the potential to serve as bio-stimulants for micropropagation in sustainable agriculture (Luziatelli et al., 2020). The potential for *Pseudomonas fluorescens* P19 and P21 to enhance root growth in olive microshoots has been documented by Peyvandi et al. (2010), who found that the root growth of olive microshoots treated with bacteria at 10^8^ CFU/mL and with L-tryptophan supplement was enhanced than when treated with synthetic IBA. Quambusch et al. (2014) found that the endophytic bacterial communities present within the different genotypes of *Prunus avium*, which were propagated with different degrees of success, differed from one another. Zamioudis et al. (2013) found that beneficial *Pseudomonas* spp. increased the capacity for root developmental plasticity. Specifically, these *Pseudomonas* spp. inhibit the elongation of the primary root while promoting the development of lateral roots, root hairs, and root branching in Arabidopsis. Yarte et al. (2022) demonstrated that certain PGPR isolated from the rhizosphere of *Handroanthus impetiginosus* improved rooting *in vitro.* PGPR strains being an alternative to the use of synthetic auxins, leading to lower plant loss during micropropagation and reduced micropropagation costs. Larraburu et al. (2007) optimized the *in vitro* propagation of Photinia with *Azospirillum brasilense* and *Azotobacter chroococcum*, respectively, accompanied by a marked increment in root formation when PGPRs were combined with auxin induction procedures (IBA), as to *A. chroococcum*´Cd for improving root/shoot ratios. This showed the benefit of using PGPRs in conjunction with auxin treatments during micropropagation. Kavino and Manoranjitham (2018) enhanced the resilience of banana micropropagation with beneficial microorganisms during *in vitro* rooting. The application of a mixture of *B. subtilis* and *Pseudomonas fluorescens* resulted in a significant decrease in the percentage of infected plants by *Fusarium* wilt, as well as an improved plant growth and yield, showing the potential utility to improve micropropagated plantlets.

Orlikowska et al. (2017) demonstrated that *Burkholderia phytofirmans* PsJN improved rooting of *Helleborus* microshoots even under less than optimal temperatures, and that IAA and ACC deaminase produced by PsJN increased rooting percentages and quality of roots. Overall, auxin-inoculated treatments had a rooting percentage of 100%. Muniz et al. (2013) demonstrated that rhizobial isolate EEL16010B, significantly enhanced *in vitro* rooting of Marubakaido apple rootstock. Comparable to synthetic indole acetic acid, this strain improved root development but showed no effect during acclimatization, indicating its use as a natural rooting promoter. Mon et al. (2021) showed that inoculating banana tissue culture plantlets with beneficial bacteria during *in vitro* rooting significantly enhanced growth parameters.

PGPMs increase the supply of important elements such as phosphorus and iron by dissolving phosphates and producing siderophores, which subsequently lead to vigorous root growth. In addition, PGPM-treated roots presented increased uptake capability and resistance to adverse environmental conditions (İpek and Eşitken, 2017; Gangwar et al., 2017; Etesami and Maheshwari, 2018; Chouhan et al., 2021).

### 3.4 Acclimatization stage

The acclimatization phase is focused on acclimating *in vitro*-grown plantlets to *ex vitro* conditions, where they are exposed to environmental stresses such as fluctuations in temperature, humidity, and light intensity. This stage is typically characterized by increased mortality rates due to the plantlets’ inability to adapt to external environmental conditions. PGPMs are associated with increased survival and growth of plantlets during the acclimatization stage. They enhance acclimatization by increasing stress tolerance via multiple mechanisms. They produce osmoprotectants (e.g., proline, trehalose), phytohormones, exopolysaccharides and ACC deaminase, to help maintain osmotic balance, limit ethylene production during stress and modulate stress-responsive pathways (Barooah et al., 2021; Patel et al., 2023; Maleki et al., 2023; Raja Gopalan et al., 2023). Also, the use of PGPMs has been shown to increase nutrient uptake, stimulate antioxidant activity and enhance vigorous root growth, therefore, increasing overall growth under the effects of abiotic and biotic stressors (Vettori et al., 2010; Panigrahi et al., 2016; Etesami and Maheshwari, 2018; Enespa et al., 2020; Cantabella et al., 2020; Michavila et al., 2022; Garai et al., 2023). Furthermore, PGPMs induce an induced systemic resistance (ISR) in plants, so that the plant will be better equipped to defend itself and decrease disease incidence when transferring *ex vitro* (Cassells and Rafferty-McArdle, 2012; Annapurna et al., 2013; Meena et al., 2020).

Orlikowska et al. (2017) reported that *Burkholderia phytofirmans* PsJN have a positive effect on the acclimatization of *Helleborus* (a cold tolerant plant), much more than previously thought. Those treated with *Burkholderia phytofirmans* PsJN and auxins had 100% success rooting, longer root length, and less acclimatization period compared to plants treated with auxins alone. Araújo et al. (2023) demonstrated that PGPB(s) reduced the acclimation period of hydroponically grown banana seedlings to a maximum range of 15–25 days. Thomas et al. (2010) examined the use of microbial inoculants during the micropropagation of tea. While *Pseudomonas fluorescens* was not able to establish *in vitro*, *Azospirillum brasilense* and *Trichoderma harzianum* overgrew cultures inhibiting the growth of shoots. Treated plantlets showed no signs of root rot, illustrating the importance of microbial assistance during the acclimatization period after plant transfer. Mon et al. (2021) demonstrated that the reintroduction of beneficial rhizospheric and endophytic bacteria, can be helpful for banana tissue culture plantlets. *Pseudomonas fluorescens*, *Bacillus putida*, *B. pumilus*, and *B. amyloliquefaciens* significantly enhanced the height of plants, leaf numbers, and the girth of their stems when inoculated *in vitro.*Michavila et al. (2022) found that the inoculation of the tissue-cultured micropropagated plantlets with *Bacillus* and *Pseudomonas* significantly improved the plantlet’s ability to survive acclimatization conditions.

Panigrahi et al. (2016) demonstrated that using a consortium of beneficial microbes, significantly improved the success of the acclimatization of tissue-cultured banana plants through the improved survival, growth, uptake, and stress tolerance of nutrients. Thus, biotization may be an effective method for overcoming acclimatization challenges in the commercial production of micropropagated plants. Rodrigues et al. (2022) showed that using *Bacillus* strains during the acclimatization of banana ‘Prata Catarina’ plants did not improve plant growth but did increase the uptake of nutrients into the banana plant. The increased uptake of nitrogen into the leaves and potassium and magnesium into the roots of the banana plant. Tytarenko et al. (2023a) reported that inoculating blackberry microclones with actinobacteria significantly improved survival and growth during acclimatization. Castro-Restrepo et al. (2022) evaluated the biotization of *Mentha* sp*icata* microplants with *Trichoderma asperellum* and *Bacillus subtilis* which resulted in 100% colonization by both endophytes and increased the survival of the microplants during acclimatization. Harish et al. (2008) demonstrated that biopriming tissue-cultured bananas with bacterial strains of *Pseudomonas* and *Bacillus* provided banana plants with systemic resistance to Banana bunchy top virus (BBTV). Indravathi and Suresh Babu (2019) reported that dual inoculation of microshoots of *Albizia amara* (a woody tree species) with *Pseudomonas fluorescens* and *Trichoderma viride* improved the survival of these microshoots to 82%. In their study, biotization improved the root and shoot growth, helping to mitigate transplant shock during the acclimatization phase of woody micropropagated plants. Jaizme-Vega et al. (2004) reported that a consortium of *Bacillus* spp. stimulated the growth and increased the levels of mineral nutrients in micropropagated bananas during acclimatization.

Kumar et al. (2014) showed that *Piriformospora indica* biotization improved both growth and anticancer activity in *Vernonia divergens.* The biotized micropropagated plants improved the shoot and root development of plants and regained significant inhibitory activity against EAC cells, thereby establishing the dual effects of fungal inoculation in relation to plant development and bioactivity.

Pandey et al. (2000) reported that tissue-cultured tea plants inoculated with antagonistic bacteria reduced fungal mortality and improved plant survival up to 100% across seasons. The treatment also effectively reduced fungal mortality from *F. oxysporum* while at the same time enhanced the growth of plants produced through both tissue culture and seed production. Rajamanickam et al. (2018) demonstrated that biohardening micropropagated banana cv. Grand Naine with *Bacillus subtilis* reduced rhizome rot disease caused by *P. carotovorum* while improving the plants’ growth and increasing the activity of two plant defense enzymes, suggesting that the bacteria ISR and improved the stability of the micropropagated plants during both primary and secondary hardening stages. Rodríguez-Romero et al. (2005) found that combined use of *Glomus manihotis* and *Bacillus* spp. led to higher growth and nutrient uptake of micropropagated banana ‘Grande Naine’ during acclimatization. Suada et al. (2015) reported that culture supernatants of *Bacillus* sp., *Pseudomonas putida* and *P. fluorescens* were effective for root and shoot growth of banana ‘Grand Naine’ during hardening. The use of *Bacillus* spp. gave a reduction of 50% to the acclimatization period, providing a significant economic advantage. Kavino et al. (2007) demonstrated that the application of the bacterial inoculants *Pseudomonas fluorescens* and *Bacillus subtilis* during the hardening process of micropropagated banana plants resulted in increased levels of defensive enzymes and reduced incidence of the BBTV. Beneficial microbes can enhance stress resistance and thus present an ecological alternative for the treatment of micropropagated bananas, compared to chemical treatments.

Moncada et al. (2015) showed that combining *Glomus* sp. with *Pseudomonas migulae* and *Bacillus amyloliquefaciens* together during blackberry micropropagation, enhanced seedling growth, root colonization and survival rate. This synergy of microbial activity enhanced the vigor of the seedlings and their acclimatization, supporting the use of arbuscular mycorrhizal fungi and plant growth promoting rhizobacteria in sustainable tissue culture. Trivedi and Pandey (2007) showed that when *Bacillus megaterium, Bacillus subtilis*, and *Pseudomonas corrugata* used together, enhanced the growth and survival of micropropagated *Picrorhiza kurrooa.* These microorganisms suppressed pathogenic fungi and improved the performance of plants in greenhouses. Vettori et al. (2010) reported that *Azospirillum brasilense* Sp245 improved growth during acclimatization of micropropagated cultivars of plum and almond-peach rootstocks, however not for apple micropropagated rootstocks. Yadav et al. (2013) found that when micropropagated *Gloriosa superba* plants were inoculated with *Acaulospora laevis*, the plant’s survival rates, root to shoot ratios, and the amount of colchicine enhanced. The combination of *A. laevis* and *Glomus mosseae* together produced higher levels of biochemical and root-leaf characteristics than either fungi (AMF) alone.

## 4 Emerging mechanisms in PGPM-mediated micropropagation

For many years, PGPMs have been recognized for their important roles in the micropropagation process, primarily related to N_2_ fixation, P-solubilization, phytohormone production and improving nutrient access through the production of siderophores. Recent research indicates that the impacts of these microorganisms may continue to extend beyond this initial impact on plant growth to include the molecular/epigenetic impacts on plants (Bond and Baulcombe, 2015; Rigal et al., 2016). Numerous PGPMs are now known to induce stable changes in DNA methylation in plant tissues by RNA-directed DNA methylation (RdDM) and thus influence plant transcription, genomic integrity and ultimately increase plant growth rates even after the removal of the PGPM(s) (Chen et al., 2022). Furthermore, PGPMs can also alter the profile of histone acetylation and deacetylation for plant tissues, which is driven by non-coding RNAs (e.g., siRNA and miRNA) during the regulation of stress-response genes (Fu et al., 2006; Kundariya et al., 2022). With the continued development of new technologies (e.g., Exo-RNAi), it will become even easier to regulate epigenetic modifications to improve micropropagation (Singh et al., 2023).

The small RNA molecules produced by PGPMs and plants form a bidirectional signalling pathway between the two organisms (Thiebaut et al., 2015; Lü et al., 2022; Middleton et al, 2024). In this regard, small RNA production regulates the plant’s immune response, growth and development pathways. Interactions between PGPMs and plants alter miRNA expression associated with root structure, and auxin and symbiotic nitrogen fixation. In contrast, some plants use miRNA to control PGPM genetic expression. The microbial tRNA-derived fragment (tRF) may influence the transition to organogenesis, which will impact the design of tissue culture systems (Ortiz Castro et al., 2012; Ren et al., 2019; Tiwari et al., 2021).

In addition to epigenetics, quorum sensing (QS) affects microbial activity and products (e.g., IAA) as well as cooperative colonization and suppression of plant pathogens (Chernin, 2011; Zaytseva et al., 2019; Ghosh and Mandal, 2022). Modifications to QS can enhance PGPM positive attributes during sensitive stages of the micropropagation process. PGPMs, such as *Bacillus* and *Pseudomonas*, stimulate induced system resistance (ISR) via the jasmonic acid and ethylene pathways by priming the plant to be more resilient to both biotic (pathogens) and abiotic (environmental) stress (Choudhary et al., 2007; Annapurna et al., 2013; Thomashow, 2016; Karthikeyan et al., 2019; Kannojia et al., 2019; Panpatte et al., 2020; Singh et al., 2024).

Thus, the current understanding of PGPMs as dynamic regulators of plant gene expression and the plant’s defense mechanisms represents a significant shift in how we utilize PGPMs during micropropagation.

## 5 PGPM-nanomaterial co-application strategies

Although the benefits of using PGPMs and nanomaterials have been demonstrated separately in micropropagation, combining these two technologies is an emerging new area of research with great potential. Applications utilizing co-application methods between PGPM and nanomaterials are emerging as a new research area that may aid plant tissue culture in overcoming previous issues related to microbial survival, nutrient delivery, and stress avoidance. Because nanomaterials can be used in conjunction with PGPMs to facilitate their use *in vitro.* By encapsulating or immobilizing beneficial microorganisms using a nanoscale carrier, the microorganisms can be more stable, controlled in their release within a tissue culture environment, and also protected from oxidative stress and osmotic shifts that normally occur in artificial media systems like MS medium, which may compromise the viability of microorganisms.

Nanomaterials have the authority to entrap signaling molecules, which can be targeted to plant tissues to enhance the activity of PGPMs (de Moraes et al., 2023). Pour et al. (2019) reported that Silica, when blended with the plant growth-promoting rhizobacteria, increased root length and shoot proliferation in pistachio micropropagation. They further reported that carbon nanotubes (CNT) facilitated microbial metabolites and improved the plant measures of growth. Given that environmental stress can limit beneficial microorganisms’ interaction, nanoscale carriers can endure and provide protective persistence in an environmental stressor context to retain viability when apply, and ultimately colonize plant tissues. With these combinations to include an effective timing of and sustained viability of microbial activity from the microbial inoculants, can promote the plant growth in a more robust methodology (Shanmugam, 2022). Much of the success of the beneficial microbes is that they can convert nutrients to bioavailable forms for the plants, nanomaterials could increase these functions from beneficial microbes to plant uptake (Yaseen et al., 2024). Nanomaterials are small enough to be absorbed into plant cells, giving opportunity for components to have direct interaction within the cell. Nanoscale carriers will improve the interactions and establishment of beneficial microbes, also noted that nanoparticles (NPs) can act as elicitors to elicit a plant defense response and support a better environment for the colonization of microbes (Singh et al., 2023).

Some specific metal and metal oxide nanomaterials have been shown to also promote PGPM metabolism and increase the degradation of the contaminants in tissue cultures, appropriately dosing and optimizing concentration (Athwal et al., 2024). Additionally, nanomaterials have the optimal size and surface area to facilitate even better interactions with microbial cells to promote impaired PGPM growth and activity toward pathogens (Sharma et al., 2024).

Using PGPMs and nanomaterials could also mitigate the problem of persistent contamination of tissue cultures. Silver nanomaterial is one metal whose antimicrobial properties are well known, can be co-dosed with PGPMs to kill or significantly reduce pathogenic bacteria and filamentous fungi, whilst allowing the commensal microbes to persist. The selective antimicrobial effects of nanomaterials provide an opportunity to limit or eliminate adding synthetic antibiotics and/or fungicides to the growth media, where relevant to tissue culture, micropropagation practices that represent a more sustainable approach. In fact, all nanomaterials potentially represent antimicrobial activity that could benefit the ability of plants grown in culture to reduce pathogens and build beneficial microbial associations (Sodhi et al., 2025).

Moreover, nanomaterials could act as nano delivery systems for the signaling molecules or secondary metabolites produced by PGPMs. As such, targeted delivery systems will allow strict control of morphogenic responses such as the timing and amount of callus, shoot differentiation, and root initiation. The purpose of these nanomaterials to improve retention of critical nutrients while in conjunction with PGPR, which in turn significantly increase the bioavailability of nutrients that are beneficial to plant growth (Kapoor et al., 2023). This would improve microbial survival in the rhizosphere, microbial activity (Ding et al., 2024), and this bio-safe delivery of nutraceuticals and growth regulators, which would to infinity optimize delivery within the plant, and continue to enhance plant-microbe symbiosis (Sodhi et al., 2025).

## 6 Synthetic biology approaches for PGPM engineering

Synthetic biology - an amalgamation of molecular biology, computational modelling and engineering - allows to engineer or edit genes and gene circuits in microorganisms with precision. These techniques have been used to increase the production of ‘plant-beneficial’ metabolites, manipulate colonization traits and improve compatibility with artificial media that we use for micropropagation systems. SCRaMbLE-in technology holds significant potential in that it allows us to rapidly prototype and integrate synthetic pathways into microbial genomes, creating PGPMs with enhanced traits (Liu et al., 2018). Constructing synthetic or recombinant DNA to augment PGPM performance will open the door to microbial formulations that meet client-specific agricultural needs (Naik et al., 2019). CRISPR/Cas9 and CRISPR interference (CRISPRi) are core synthetic biology strategies for the manipulation of growth-promoting microorganisms. These methods enable the targeted engineering of genomes and microbes that favour greater microbial colonization or have improved functioning in the optimum metabolic pathway in the PGPM-micropropagation process for sustainable agriculture (Binodh et al., 2024). Using synthetic biology techniques to alter metabolic pathways also provides successful improvements in stress tolerance and plant resilience (Fan, 2024).

The idea of regulating activity in PGPMs is a dynamic approach to application that has not been previously attempted in their use, allowing them to be engineered to respond to real-time variability in the plant culture. PGPMs operate as live agents that will respond to the growth status of the host plant, with microorganisms like strains of *Bacillus* or strains of *Cryptococcus* have been shown to improve plant parameters like biomass and stem height when plants experience saline stress (Arminjon and Lefort, 2025).

Engineering of stress-responsive promoters such as the *ect promoter* will also provide enhanced transcription response of osmostress to osmotic stress. Even site-directed mutagenesis of promoter regions to increase their sensitivity and strength, and ultimately produce stress-related proteins by expression of osmotic stress genes, even at mild osmotic stress conditions (Czech et al., 2018).

Genetic engineering can also be used to change the expression of proteins involved in different stress response pathways. For instance, *Pseudomonas fluorescens* gave a highly diverse protein expression response under osmotic stress, responding, the induced osmostress response produced proteins involved in osmoprotectant production, as well as DNA repair mechanisms, and could also undergo genetic engineering and scientific optimization (Ashwitha et al., 2018). Increased growth and plant growth-promoting activities under osmotic stress have been observed in strains of *Bacillus* sp. and *Bacillus cereus*, indicating a favorite target for some level of genetic modification to maximize these traits to develop bioinoculants for drylands (Bandeppa et al., 2018).

The principles of synthetic biology include modular design. Synthetic biologists can make functional units, which can be assembled to create complex systems. Computational models can enable synthetic biologists to predict how interactions and behaviors change among these microbial communities as we design them through modeling the different interactions before we conduct these systems (Matuszyńska et al., 2024). DNA synthesis and protein engineering allow for the development of synthetic circuits to govern microbial interactions, which enhance cooperation and division of labor in each microbial community (León-Buitimea et al., 2022; Zhang et al., 2023). The bioengineered interactions provide more stability and efficiency in synthetic microbial communities, allowing them to be utilized in applications such as bioremediation and biosynthesis of valuable compounds (Bai et al., 2024).

## 7 Multi-omics approaches in unravelling complex plant-PGPM interactions

The combined use of multiple omics techniques has provided researchers with a significantly enhanced understanding of how plant-associated microorganisms promote plant growth. These techniques include genomics, transcriptomics, proteomics, metabolomics, and metagenomics, providing researchers with various perspectives on the complex molecular communications that occur between these groups of organisms when they interact (Mmotla et al., 2025).

Researchers can use genomics to identify genes in both plants and microorganisms that are involved in promoting plant growth or responding to environmental stressors, thereby enabling the development of bio-inoculants (Salvadi and Mir, 2024). The field of metagenomics enables researchers to characterize the diversity of and potential functional roles played by microbial communities found in the rhizosphere, and how these communities contribute to nutrient cycling and plant health (Pal and Sengupta, 2022). Researchers studying the Transcriptome can identify gene expression profiles during interactions between plants and microorganisms, which highlights the major molecular pathways that regulate symbiosis and plant resilience (Salvadi and Mir, 2024; Bhimani et al., 2024). Metatranscriptomics examines how the active microbial community contributes to their activity in various phases of interaction with plants (Pal and Sengupta, 2022). Proteomics will identify which proteins are found in the signal transduction and metabolic pathways related to plant-microorganism interactions, and is another vital component of our understanding of their symbiotic relationship (Kaur et al., 2022; Bhimani et al., 2024). The metabolome is analysed to identify the plant’s metabolites that may be used to combat disease or promote the growth of the plant and to develop a target base to maximize crop yield (Salvadi and Mir, 2024).

While multi-omics gives us a comprehensive view of how PGPMs interact with their host plants, traditional methods can be implemented to verify the results of multi-omics studies, and to provide a full examination of specific interactions under more controlled laboratory conditions. A combination of traditional and modern techniques is required for the evolution of sustainable agricultural practices.

## 8 Challenges and limitations of utilizing PGPMs in micropropagation

PGPMs have multiple benefits in improving the efficiency and sustainability of micropropagation, but their incorporation into the plant tissue culture system poses several challenges. To enable the broad-scale application of PGPMs, however, important technical, biological, and operational challenges need to be overcome.

One of the key problems with the use of these microorganisms is their natural host specificity. The efficacy of a certain PGPM strain is often specific to the host plant species. Although certain microorganisms have broad-spectrum effects, many strains are highly specific and do not lead to desirable effects on all plant species or cultivars (Drogue et al., 2012; Mahadevakumar and Sridhar, 2020).

PGPM plant–microbe interactions also differ widely and are shaped by the plant genotype, microbial strain, and environmental conditions. This variation poses challenges to generalizing their application across different species. The specificity of PGPMs requires the design and development of customized microbial formulations designed for each plant species, which increases the time and cost of the design and study of PGPMs for industrial use. This challenge can be addressed through careful screening and selection of PGPM strains tailored to the specific plant species of interest, an effort that is nevertheless often time-consuming (Khatoon et al., 2022; Muñoz-Carvajal et al., 2023).

A major challenge is the inconsistent performance of PGPMs across diverse conditions. Although PGPMs may demonstrate initial success under laboratory conditions, tropes of these products often lose efficiency when field or commercial-scale conditions come into play. Their performance can be notably affected by temperature, humidity and soil composition. It has been shown that some strains of PGPMs are more sensitive to abiotic stress, such as temperature fluctuations, and become less effective in less-than-optimal growing conditions (Khare and Arora, 2015; Moore et al., 2023). In addition to the effects of micropropagation conditions, as the composition of the culture media varies, PGPM activity can also be hindered, as the presence of sugar at high concentrations negatively affects the performance of some strains of PGPMs (Kadner et al., 1992; Yorek et al., 1993).

During acclimatization, when PGPM-treated plantlets are transferred to soil, the introduced microorganisms compete with indigenous soil microorganisms. Thus, one consequence of their competitive dynamics is a reduction in populations and activity of the microorganisms in the long term, which limits the known benefits stemming from these mutualistic associations (Musarrat and Khan, 2014).

These discrepancies underscore the need for extensive experimentation in numerous environments to assess the consistent efficacy of PGPMs in different habitats. These microorganisms are often included in micropropagation systems to stimulate the growth of plants; however, their use poses a risk of contamination, especially in sterile tissue culture environments.

PGPM preparations may involve microbes that can jeopardize the sterility of the tissue culture environment (Orlikowska and Zawadzka, 2006). In large-scale applications, their presence in culture media increases the likelihood of cross-contamination between culture vessels (Sharaf-Eldin and Weathers, 2006). In some cases, specific strains can generate metabolites that are detrimental to plant tissues, which inhibit growth and/or cause tissue necrosis (Åström et al., 1993). The use of these microorganisms is challenging because one needs to balance the two opposing needs of promoting plant growth while keeping the tissue culture system sterile.

Although the possible advantages of using PGPMs are well understood, the actual mechanisms of action associated with their efficacy in micropropagation systems are poorly understood. This uncertainty inhibits the development of protocols to optimally apply these growth-promoting microorganisms. The interactions of PGPMs with plants and their environmental conditions have complex biochemical and other molecular bases that are not fully understood. Allocating research to understand the mechanisms of action of PGPMs for micropropagation systems would benefit these systems so that these microorganisms can be better applied and prove to be a more reliable component of micropropagation. Transferring PGPM usage from the laboratory level to successful implementation in commercial systems for micropropagation has unique challenges (Takors, 2016). First, large-scale production will require special equipment and facilities to maintain the viability and functionality of the microorganism over periods of time for use as PGPMs in micropropagation systems. The stability of their formulations for use is a restricting issue for commercial use, as many strains of PGPMs have relatively low viability and can be used in the future for effective application as systematic resistance-generating methods or targets. Furthermore, while some may be able to produce these microorganisms as products for definitive usage in commercial production systems, there is a lack of established protocols for their application and operational usage. The operational usage ranges from specific dosage, timing of application, and method of application, which will all potentially have variable effects. Inevitably, replicating previously achieved results on a grander scale comes with inherent challenges (Joshi, 2023). The use of PGPMs for micropropagation raises additional regulatory and biosafety concerns, especially in regions with strict agricultural and environmental regulations (Glick, 2015). The introduction of nonindigenous strains of PGPMs into local ecosystems can disrupt native biodiversity. Approving commercial plant growth-promoting microorganism products can be time-consuming and costly because safety and efficacy testing are often required and must be reproduced in relevant agricultural setting(s). Additionally, public perceptions of microbial products can inhibit acceptance. There may be consumer hesitancy to adopt plantlets inoculated with microorganisms; this is particularly possible if they have not encountered PGPMs before this experience. Through a combination of meeting regulatory requirements and public outreach efforts, these issues must be addressed to facilitate their successful commercialization (Selvakumar et al., 2013; Vílchez et al., 2016; Sundh et al., 2021).

Incorporating PGPMs into micropropagation functions requires a considerable investment in upfront capital, which can be a difficulty for small-scale agricultural producers. The screening, selection, and testing of different plant growth-promoting microorganism strains require significant R&D investments, and introducing PGPMs into micropropagation typically means that existing infrastructure will need to be modified, for example, through the addition of bioreactors for their production (Hvoslef-Eide et al., 2003; Honda and Kobayashi, 2004). The value of PGPMs relies on the availability of trained individuals who are knowledgeable about microbiology and plant tissue culture methods. As additional staff training and education are required to cover this lapse in knowledge, the cost of implementation increases (Muñoz-Carvajal et al., 2023). The costs of PGPMs undoubtedly contribute to the overall expenditures of micropropagation; however, the long-term advantages help compensate for these costs, for example, through decreased reliance on chemical inputs and increased survival rates of plantlets.

Most of the research into PGPMs in micropropagation has focused on only a limited number of crops, and this limited research path regarding certain plant species limits the generalizability of the broader concept of these microorganisms in relation to other economically important plants or plants of regional importance. Although there is a need to study their response in commercially important crops, such as medicinal plants or ornamental species, research on this topic is still lacking. The efficacy of these microorganisms may also differ within each geographical area because of differences in factors such as the soil microbial community, climate, or crop diversity. Research that is specifically designed for each region is important for identifying surrounding issues. There is a need to expand research on PGPMs to more crops and geographical areas to realize their potential in micropropagation.

## 9 Future perspectives on the utilization of PGPMs in micropropagation

The incorporation of PGPMs in micropropagation represents a significant breakthrough in sustainable plant tissue culture. Considerable progress has already been made in this respect, and there is still much scope to further enhance the application of PGPMs in tissue culture systems through technology diversification, interdisciplinary approaches, and commercialization. In the future, challenges that must be overcome while expanding the role and effectiveness of these microorganisms in micropropagation systems must be addressed.

One major area for future research concerns the development of crop-specific PGPMs. Owing to the highly targeted nature of PGPM–plant interactions, future studies should focus on the isolation and characterization of strains that have been adapted to particular plant species or cultivars. Advances in genomics and transcriptomics could be used to understand plant-PGPM molecular interactions. This approach will help identify compatible strains with maximal growth-promoting effects on certain crops. Although most ongoing investigations are limited to single microbial species, future studies may involve the establishment of consortia of competing PGPM types. These consortia may combine the benefits of bacteria, fungi, and actinomycetes, thus facilitating different modes of plant growth promotion and stress tolerance. We believe that the design of crop-specific PGPM formulations has the potential to substantially improve the reproducibility and efficacy of micropropagation systems, especially for high-value crops such as medicinal, ornamental and fruit plants.

PGPMs and PGPM-enriched microbial consortia offer several promising biotechnological opportunities. Their integration into widely used micropropagation workflows could open new possibilities for future tissue-culture-based propagation systems. Such innovative tools may further support the understanding, design, and use of PGPMs. Synthetic biology approaches can be used to develop PGPMs with desired characteristics, such as increased phytohormone production, increased tolerance to abiotic stress, or resilience to changes in environmental conditions. Optimized, engineered PGPMs can exhibit robust functions under different culture conditions. In the era of nanotechnology, the purpose of the current review is to highlight the potential of PGPM nano-based formulations to improve microbial delivery and stability in tissue culture systems. PGPMs can be protected from adverse environmental conditions via encapsulation techniques using biopolymers or NPs, and prolonged release into the culture medium can be achieved. Bioinformatics and machine learning-based predictive models can mine large datasets to discern the best-performing PGPMs for specific crops or culture conditions. These technologies could also predict the best dosage, timing, and combinations of PGPMs to elicit maximal efficacy. These tools help PGPMs achieve stellar performance while reducing the time cost and tone associated with the development and implementation of PGPMs.

Hence, future studies might focus on optimizing the delivery system of these microorganisms in micropropagation systems to ensure their colonization and functional activity in plant tissue. Murashige and Skoog (MS) media and several other culture media could be enhanced for more efficient propagation and functional activity of PGPMs. In various systems, the secretion of PGPMs can be blunted by immobilizing them on substrates such as alginate beads or biopolymer matrices that allow regulated release into culture systems, reducing the chance of overgrowth and increasing sterility.

By treating explants with PGPMs before the inoculation of tissue culture media, microbial colonization can be promoted for better plant–microbe interactions at the beginning of the micropropagation stages. This will enhance the compatibility and efficacy of PGPMs when used in tissue culture but also ensure that sterility and precision, both of which are integral to the success of micropropagation, are maintained.

Most of the studies on PGPMs in micropropagation have focused mainly on widely cultivated species, and it is imperative to broaden research efforts to encompass lesser-studied plant taxa. These microorganisms in the micropropagation of medicinal plants can increase the production of bioactive metabolites and in-process sustainable systems. They can increase the *in vitro* propagation and stress mitigation efforts of threatened and rare plant species during the acclimatization phase. Valuable ornamental flora and forestry species will also benefit from improved rooting, shoot proliferation, and acclimatization processes. These research efforts will promote biodiversity and enable the emergence of niche markets around these microorganisms.

In future research related to PGPMs and micropropagation, much of the work will focus on the investigation of relevant microbial metabolites and VOCs that can promote plant growth and morphogenesis *in vitro* conditions. Microbial metabolites may support reductions in the use of synthetic hormones, improvement of regeneration and rooting, and contribute to reductions in plant stress or contamination. Developments in metabolomics and synthetic biology will facilitate precise modifications of these bioactive products to support sustainable and biologically modifiable micropropagation systems.

For micropropagation with PGPMs to be commercially viable, issues of scalability and economic feasibility need to be resolved. Cost-effective approaches to mass-producing PGPMs with extended shelf-life should be pursued first. These approaches could include bioreactor systems, freeze-drying systems, and carrier systems. Standardized deployment protocols for PGPMs in micropropagation systems across different types will increase dependability. Such protocols must specify the dosage, timing, and application procedures. Commercial tissue culture systems require PGPMs to be incorporated seamlessly without major infrastructure or operational changes. Their successful integration into commercial micropropagation depends on addressing these factors, which will also enable their widespread adoption throughout the plant biotechnology industry.

The secure and sustainable application of these microorganisms in micropropagation requires systematic resolution of biosafety and regulatory challenges. It is essential to perform thorough environmental assessments to determine how they affect native ecosystems and their associated risks. The development of transparent and uniform regulatory systems to manage the approval process and market introduction of PGPM products will encourage their use and protect both safety and quality benchmarks. Providing information about benefits and safety profiles to the public will help overcome doubts and promote acceptance of these innovations in agricultural and horticultural fields. Maintaining biosafety and regulatory standards will establish stakeholder trust and enable broader adoption of PGPM in tissue culture systems.

Researchers need to explore how PGPMs interact with sustainable technologies to maximize their benefits. When these amendments are combined with biochar or organic amendments, they show promise for improving soil health while increasing the acclimatization of micropropagated plantlets. Green-synthesized NPs combined with PGPMs increase the ability of plants to absorb nutrients and withstand stress. These microorganisms can be integrated into pest and disease management approaches that help reduce the use of synthetic pesticides and fungicides in micropropagation systems. The combination of these methodologies will enable the development of complete sustainable systems for plant production.

## 10 Conclusion

A sustainable approach and a novel plant tissue culture line of research to improve micropropagation protocols is the inclusion of microbial inoculants in micropropagation systems. PGPMs have been proven to increase the efficiency of micropropagation, as they stimulate explant establishment, enhance shoot and root morphology, assist in nutrient uptake, and regulate acclimatization. In addition, these microorganisms may help to reduce the dependence on synthetic chemical inputs, thus making micropropagation environmentally sustainable and economically feasible. Despite their enormous potential, microbial specificity, contamination concerns, variable performance, and regulatory hurdles need to be addressed to enable more widespread use. Some promising routes to enhance the efficacy of PGPM applications are due to advances in biotechnological approaches, including genetic engineering, bioinformatics, and nanotechnology. Future efforts should be directed toward the development of strategies for high-throughput generation and the formulation of protocols and policies that will allow for the safe and effective deployment of PGPMs. With the increasing global need for sustainable agriculture, innovations based on these microorganisms will serve to satisfy this need while saving resources and reducing environmental impacts. The use of these microorganisms is an important step in the development of more sustainable and robust systems for plant production.
